# News and Miscellany

**Published:** 1900-11

**Authors:** 


### News and Miscellany.

---

**New subscribers** who send us $.100 now, will receive November and Decembei numbers free.

---

**Dr. A. E. McMahon,** of Tolar, Texas, formerly of Marshall, Texas, died at Knolle, Texas, June 13, 1900.

---

**Dr. Menger's** illustrated paper on the mosquito, which appeared in our October (ult.) number, will be sent to anyone on request if a two-cent stamp is enclosed. Address, Dr. R. Menger, San Antonio, Texas.

---

**Dr. J. E. Howze** has located in Austin for the practice of his specialty, eye, ear, nose and throat diseases. Dr. Howze has just returned from Europe, where he completed his studies at Berlin, Vienna and other great clinics.

---

**The situation** at Galveston has so improved that the State Board of Health, which has been in almost continuous session, adjourned indefinately September 21.—*Journal American Medical Association.*

The editor of the *Journal of the American Medical Association* is hardly excusable for not knowing that Texas *has no* "*State Board of Health*" and never had! The medical profession of the State have been trying for twenty years to get a State Board of Health, and are still trying.

---

**New Orleans Polyclinic.**—Physicians will find the Polyclinic an excellent means for posting themselves upon modern progress in all branches of medicine and surgery. The specialties are fully taught, particularly laboratory work. Fourteenth annual session opens November 12, 1900. For further information address Dr. Isadore Dyer, Secretary, New Orleans Polyclinic, New Orleans.

---

**What It Is.**—There are some "osteopaths" in Austin. In the daily papers they have an ad in which they give the following lucid explanation of the science of osteopathy: "It is a system of treating diseases without drugs, by the correct adjustment of the bones and their attachments and through them other parts of the body, in order that all parts may perform their functions in harmony. The object of osteopathic treatment is to establish the freedom of action of all fluids, forces or substances pertaining to life."

---

**By the will** of the late Dr. J. M. Da Costa, formerly professor of the practice of medicine and of clinical medicine in Jefferson Medical College, the sum of $5,000 is devised to the Pennsylvania Hospital for the endowment of a free bed, in memory of his son, John M. Da Costa; $5,000 to the College of Physicians of Philadelphia, for the endowment of a publication fund; $5,000 to the University of Pennsylvania for the professors' retiring fund. His valuable and extensive medical museum, including charts, models, medical pictures, and drawings, is bequeathed to Jefferson Medical College. His medical library, containing many good books, is bequeathed to the College of Physicians of Philadelphia.

---

**A Good Chance for a Good Doctor.**—A long established and paying practice, netting about $3500 a year, in the county seat of one of the best farming counties in Southern Texas, not very far from San Antonio, Austin, Houston, a section above overflow and out of reach of the waves, can be secured by purchasing the residence of the advertiser. Residence cost $3500 and is in good condition. County thickly settled; mostly German farmers and stock raisers. Town has about 2,000 inhabitants. Good society, schools, and all requirements of a desirable place to reside. Advertiser is a well known physician. He is actuated by the necessity of seeking a higher and dryer climate. Refers to the editors of this journal, Austin, Texas, to whom all replies should be addressed. (Mark letters "B.")

---

**W. B. Saunders & Co.** desire to announce that they are about to establish a branch of their business in Great Britain. Mr. Saunders has recently spent several weeks in London, where all the arrangements preliminary to the opening of an English house have been completed. This London branch will be operated in immediate connection with the home establishment, and the same methods that have been so successful in building up the business in this country will be employed in the conduct of this new branch. The details of the various departments of the firm's affairs have now been developed to such a state of perfection that the house feels the time has come for extending its field of operations. For a number of years Saunders' books have been sold in England through the agency of a London publisher, and, although they have already met with remarkable favor, the house is confident that by applying to the English market the same policy that has proved so successful at home, the sale of its publications in Great Britain and her colonies can be enormously increased.

---

**Do not fret** over uncertainties and disappointments. They are inevitable.

The chickens fattened for the priest
  Are captured by the weasels.
The children booked for whooping-cough
  Are taken down with measles.

"When weariness with life your spirit fills,
  When deep disgust consumes you with your lot,
Draw some store of comfort from the ills
  *You haven't got.*

"The common woes of life are bad enough;
  Misfortunes fall as easy as the dew;
And still for every morning's steak that's tough,
  *There might be two.*

"Oh trust me—better not make ado
  At the few miseries of our common lot;
There's millions of 'em—if you only knew—
  *You haven't got.*"

—Dr. Reid, in *Medical Gleaner.*

---

### Clubbing Rates.

We offer in connection with the Journal, Miss Giberne's book, "Story of the Sun, Moon and Stars" (see book notices) and Dr. Daniel's book, "Recollections of a Rebel Surgeon," at the following club rates:

| | |
|---|---|
| Texas Medical Journal, one year | $1.00 |
| Sun, Moon and Stars, $1.50; postage, 17c | 1.67 |
| | $2.67 |

Both for $1.75. Cash with order.

| | |
|---|---|
| Texas Medical Journal, one year | $1.00 |
| Recollections of a Rebel Surgeon, postpaid | 1.10 |
| | $2.10 |

Both for $1.75. Cash with order.

| | |
|---|---|
| Texas Medical Journal, one year | $1.00 |
| Recollections of a Rebel Surgeon, postpaid | 1.10 |
| Sun, Moon and Stars, postpaid | 1.67 |
| | $3.77 |

The three for $2.50. Cash with order.

